# Experimental Models for Investigating Intra-Stromal Migration of Corneal Keratocytes, Fibroblasts and Myofibroblasts

**DOI:** 10.3390/jfb3010183

**Published:** 2012-03-19

**Authors:** Walter Matthew Petroll, Neema Lakshman, Lisha Ma

**Affiliations:** Department of Ophthalmology, University of Texas Southwestern Medical Center, 5323 Harry Hines Boulevard, Dallas, TX 75390, USA

**Keywords:** extracellular matrix, corneal keratocytes, cell mechanics, growth factors, 3D culture

## Abstract

Following laser vision correction, corneal keratocytes must repopulate areas of cell loss by migrating through the intact corneal stroma, and this can impact corneal shape and transparency. In this study, we evaluate 3D culture models for simulating this process *in vitro*. Buttons (8 mm diameter) were first punched out of keratocyte populated compressed collagen matrices, exposed to a 3 mm diameter freeze injury, and cultured in serum-free media (basal media) or media supplemented with 10% FBS, TGFβ1 or PDGF BB. Following freeze injury, a region of cell death was observed in the center of the constructs. Although cells readily migrated *on top* of the matrices to cover the wound area, a limited amount of cell migration was observed *within* the constructs. We next developed a novel “sandwich” model, which better mimics the native lamellar architecture of the cornea. Using this model, significant migration was observed under all conditions studied. In both models, cells in TGFβ and 10% FBS developed stress fibers; whereas cells in PDGF were more dendritic. PDGF stimulated the most inter-lamellar migration in the sandwich construct. Overall, these models provide insights into the complex interplay between growth factors, cell mechanical phenotypes and the structural properties of the ECM.

## 1. Introduction

The cornea is an optically clear tissue that forms the front surface of the eye, and accounts for nearly two-thirds of its refractive power. The corneal stroma, which makes up 90% of corneal thickness, is a highly ordered structure consisting of approximately 200 collagen lamellae [1]. Corneal stromal cells (keratocytes) reside between the collagen lamellae, and are responsible for secreting ECM components required to maintain normal corneal structure and function. From a mechanical standpoint, resting keratocytes are considered quiescent; they do not express stress fibers or generate substantial contractile forces [2,3]. However, following injury, quiescent corneal keratocytes surrounding the wound generally become activated, and transform into a fibroblastic repair phenotype [4,5]. In certain wound types, fibroblasts further differentiate into myofibroblasts, which are associated with scar formation [6,7].

Because of its accessibility and optical power, the cornea is the target for numerous refractive surgical procedures. Modern laser vision correction procedures, such as photorefractive keratectomy (PRK) and laser-assisted *in situ* keratomileusis (LASIK), reshape the corneal stroma using photoablation in order to achieve a desired change in refractive power. Both of these procedures induce keratocyte death in stromal tissue surrounding the area of photoablation [8]. While repopulation of these regions is desirable, the normal wound healing response can lead to fibrosis and scarring along the visual axis in a subset of patients, especially following PRK [9]. This can cause a permanent reduction in corneal clarity and a can also decrease the refractive effect of the surgery [9,10]. Thus from a clinical standpoint, it is preferable to minimize cellular force generation and fibrosis during stromal repopulation. Such non-disruptive stromal repopulation is also needed following UV cross-linking of the cornea, which is increasingly used as a treatment for keratoconus, since this procedure kills corneal keratocytes in the area of treatment [11,12]. Despite the clinical importance of intrastromal wound healing, *in vitro* models for simulating this process are limited. 

Numerous cell culture studies have identified several key growth factors that play an important role in mediating keratocyte differentiation into specific wound healing phenotypes [6,7,13,14,15,16,17,18,19,20,21,22,23]. However, these studies have been performed primarily using 2-D substrates, and keratocytes reside within a complex 3-D extracellular matrix *in vivo*. Significant differences in cell morphology, adhesion organization, and mechanical behavior have been identified between 2-D and 3-D culture models, with 3-D models more closely mimicking *in vivo* cell behavior [24,25,26,27,28,29,30,31,32]. We previously established that corneal keratocytes are able to differentiate normally and respond to growth factors within compressed collagen matrices, which provide a high-stiffness, 3D environment, similar to native stromal tissue [33,34]. In this study, we investigate whether compressed collagen matrices can be used as a novel platform for investigating keratocyte behavior during intrastromal wound healing.

## 2. Experimental Section

### 2.1. Cell Culture

Corneal keratocytes were isolated from rabbit eyes obtained from Pel Freez (Rogers, AR, USA) as previously described. Cells were cultured in tissue culture flasks with medium consisting of Dulbecco’s modified Eagle’s minimum essential medium with pyruvate (DMEM; Invtrogen, Carlsbad, CA, USA), supplemented with 1% RPMI vitamin mix (Sigma-Aldrich, St. Louis, MO, USA), 100 μM nonessential amino acids (Invitrogen, Carlsbad, CA, USA), 100 μg/mL ascorbic acid, and 1% penicillin/streptomycin amphotericin B (Fungizone; BioWhittaker, Inc., Walkersville, MD, USA) to maintain the keratocyte phenotype.

### 2.2. Preparation of Cell-Seeded Compressed Collagen Matrices

To prepare compressed collagen matrices, 10 mg/mL of Type I Rat Tail collagen (BD Biosciences, San Jose, CA, USA) was diluted to a final concentration of 4 mg/mL, 0.6 mL of 10X DMEM was added to 5 mL of the collagen solution and after drop-wise neutralization with 1N sodium hydroxide, a suspension of 8 × 10^6^ keratocytes in 0.6 mL serum-free media was added to the collagen mixture. The solution containing cells and the collagen was poured into a 3 × 2 × 1 cm well and allowed to set for 30 min at 37 °C. Following polymerization, the matrix was compacted by external compression as previously described [34,35]. Briefly, a layer of nylon mesh (~50 µm mesh size) was placed on a double layer of filter paper. The matrices were placed on the nylon mesh, covered with another layer of nylon mesh and a pane of glass, and loaded with a 130 g stainless steel block for 5 min at room temperature. This led to the formation of flat collagen sheet approximately 400 microns thick, with a cell density of ~33,000 cells/mm^3^, which is somewhat higher than that of the human cornea (20,522 ± 2,981 cells/mm^3^) [36].

### 2.3. Preparation of Sandwiched Compressed Collagen Matrices

To prepare compressed sandwich collagen matrices, 0.3 mL of 10X DMEM was added to 2.5 mL of collagen solution and after drop-wise neutralization with 1N sodium hydroxide, 0.3 mL serum-free media was added to the collagen mixture. The collagen mixture with no cells was poured into a 3 × 2 × 1 cm well and allowed to set for 30 min at 37 °C. Following polymerization, a suspension of 2 × 10^6^ or 5 × 10^5^ keratocytes in 1 mL serum-free media was poured over the polymerized collagen. After 30 min to allow cell attachment to the matrix, media (including unattached cells) was removed from the top of the collagen. Another layer of collagen was poured over the layer of cells and incubated for 30 min at 37 °C. Following polymerization, the matrix was compacted by external compression as described above.

### 2.4. *In Vitro* Injuries

Following compression, 8mm diameter buttons were punched out of the compressed matrix and incubated in serum-free media for 24 hours to allow cell spreading. We then simulated an injury by either: (1) immersing a 3 mm diameter cylindrical stainless steel probe (25 g) with a polished flat tip in liquid nitrogen for 20 seconds, then placing the tip against the surface of the construct for 5 seconds using only gravitational force (Freeze injury), or (2) pushing on the surface near the center of the matrices for approximately 1 second using either a 3 mm diameter flat tip probe or a glass probe with a 5 mm diameter spherical tip (Mechanical Injury). Following injury, the buttons were then cultured in serum-free media supplemented with either 10% FBS, TGFβ1 (10 ng/mL, Sigma Aldrich, St. Louis, MO, USA), PDGF BB (50 ng/mL, Millipore, Temecula, CA), or no growth factor (control) for up to 4 days. Growth factor concentrations were determined from previous studies, and represent the lowest concentration to give a maximal effect [37].

### 2.5. Live/Dead Staining

To determine the effects of injury simulation, the Live/Dead® Viability/Cytotoxicity Assay Kit (Invitrogen; CA) was used. This assay is based on the simultaneous determination of live and dead cells with two probes that measure recognized parameters of cell viability—intracellular esterase activity and plasma membrane integrity. One day after *in vitro* injury to the compressed matrix buttons, LIVE/DEAD^®^ labeling was performed according to the manufacturer’s instructions. Labeled cells were then imaged using confocal microscopy. 

### 2.6. F-Actin and DNA Labeling

One to seven days after injury, constructs were fixed using 3% paraformaldehyde in phosphate buffer for 10 min and permeabilized with 0.5% Triton X-100 in phosphate buffer for 3 min. Cells were labeled with Alexa Fluor 488 Phalloidin (1:50; Molecular Probes, Eugene, OR, USA) for 1 h and then washed in phosphate buffer saline (PBS; 3 times for 5 min). To stain the cell nuclei, TOTO-3 iodide (1:200; Molecular Probes, Eugene, OR, USA) was then added to each sample for 15 min, and samples were washed a final time with PBS.

### 2.7. Laser Confocal Microscopy

**Figure 1:**
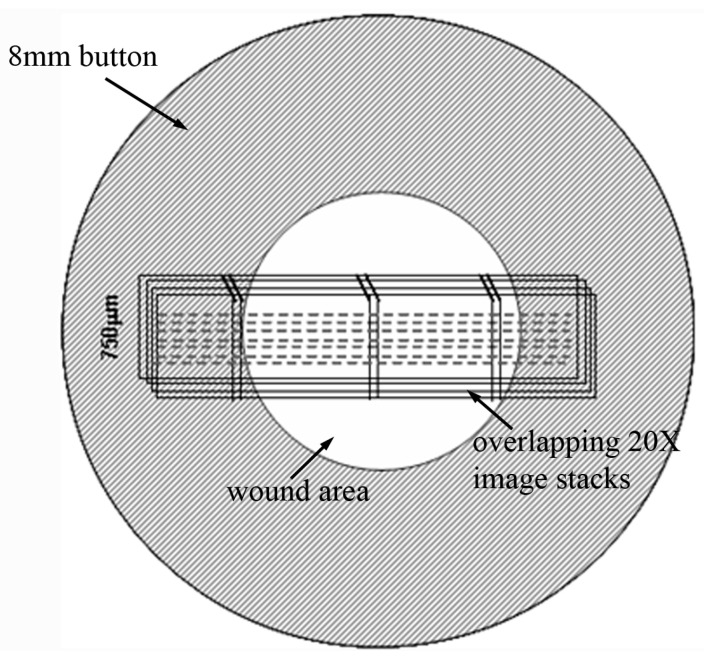
Schematic showing where the montage of 3-D image stacks was collected for each sample.

After labeling the cells, fluorescent (for f-actin and nuclei) and reflected light (for collagen) 3-D optical section images were acquired simultaneously using laser confocal microscopy (Leica SP2, Heidelberg, Germany). Stacks of optical sections (z-series) were acquired by changing the position of the focal plane in 4 μm steps for a 20× objective (non-immersion, 0.7 NA, 590 μm free working distance) or 1μm steps for a 63× objective (water immersion, 1.2 NA, 220 μm free working distance). Overalapping image stacks were collected across the entire wound area, as shown in Figure 1.

### 2.8. Assessment of Cell Migration

Maximum intensity projections of the F-actin and TOTO-3 image stacks were created, and color overlays were generated using Metamorph (Molecular Devices, Sunnyvale, CA, USA). Photoshop (Adobe, Mountain View, CA, USA) was then used to align the overlapped images to create a montage of the injured area. Each montage included the wound area along with border region outside the injury. The distance that cells had traveled into the wound area was calculated by drawing a straight line between the edge of the injury and cells at the leading edge of the migratory front. 

## 3. Results and Discussion

We recently developed a model for directly investigating cell-matrix mechanical interactions during migration in which cell-seeded compressed collagen matrices are nested within acellular uncompressed matrices [33,38]. This model simulates the process of keratocyte migration out of the rigid corneal stroma and into a loose provisional matrix, as might be observed following injury or incisional surgery. Using strip testing, the elastic modulus of compressed matrices has been reported to be approximately 250 MPa [39]. Furthermore, since quiescent (non-contractile) cells can be used in the inner matrix, the effects of specific growth factors can be assessed without pre-exposure to serum or other factors which may permanently alter these responses [20]. In this study, we investigated whether compressed collagen matrices can be used as a novel platform for investigating keratocyte behavior during *intrastromal* wound healing. We used both standard cell-seeded compressed collagen matrices, and a novel sandwiched matrix construct which better mimics the architecture of the native cornea.

**Figure 2:**
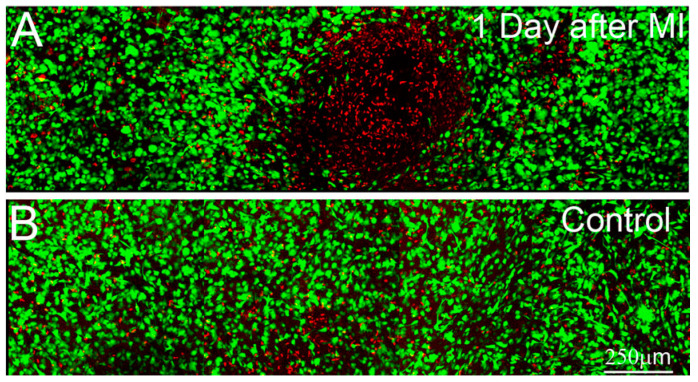
Maximum intensity projection images (~50 microns thick) of Live/Dead staining after 1 day of culture in 10% FBS. Live cells are labeled green and dead cells are labeled red. (**A**) 1 day after mechanical injury (MI), induced by pushing on the surface of the matrix using a probe with a 5mm diameter spherical tip; (**B**) 1 day control sample, which was left untouched.

### 3.1. Standard Compressed Collagen Matrices

To evaluate the effects of different experimental wounds (e.g., mechanical injury, freeze injury) on cells within compressed collagen matrices, live-dead staining was used. Mechanical injury was first attempted by pushing on the surface near the center of the matrices for approximately 1 second using a 3 mm diameter stainless steel probe with a flat tip. Using this approach, cell death was limited to isolated regions at the edges of the area of probe application (not shown), where stress concentrations and large shear strain would be expected. Furthermore, cell death was only observed in cases where significant force was applied, which resulted in deep indentations (100 to 200 μm) on the top of the construct. We also attempted the procedure using a 5 mm diameter rounded glass probe, which would be expected to provide a more uniform shear strain. Using this approach, a region of cell death was observed in the center of the constructs, whereas the surrounding cells remained viable (Figure 2). However, significant pressure was again needed to produce any sort of injury, which resulted in significant distortion and indentation of the compressed ECM. Similar issues were encountered using smaller diameter probes. Overall, inducing cell death via mechanical injury was difficult and produced significant distortions of the construct, thus this approach was not pursued further.

**Figure 3:**
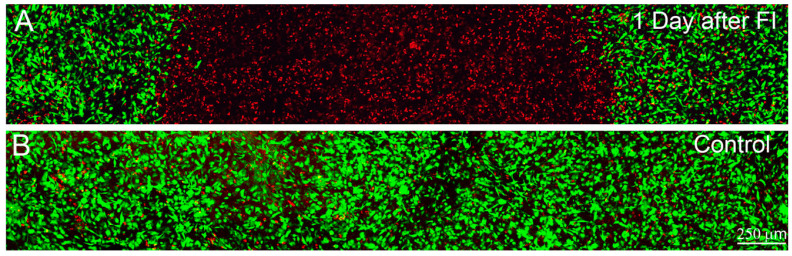
Maximum intensity projection images (~50 microns thick) of Live/Dead staining in standard compressed matrices after 1 day of culture in 10% FBS. Live cells are labeled green and dead cells are labeled red. (**A**) 1 day after freeze injury (FI), induced by pushing on the surface of the matrix using a cold 3 mm diameter probe; (**B**) 1 day control sample, in which a room temperature probe was used.

We next attempted to induce a region of cell death by using a cryo injury. Transcorneal freeze injuries have been used previously in animal studies to produce a full-thickness region of cell death within the central cornea [40,41,42]. This is generally accomplished by placing a stainless steel probe in liquid nitrogen, then holding it against the anterior corneal surface for several seconds. Similarly, freeze injury was induced *in vitro* by placing the cold tip of a flat 3 mm diameter probe on the surface of the compressed collagen matrices, using only gravitational force. Following freeze injury a region of cell death was observed in the center of the constructs, whereas the surrounding cells remained viable (Figure 3A). Cell death was observed throughout the full thickness of the construct. However, the diameter of this region was maximal at the top of the construct and gradually decreased with depth, consistent with *in vivo* studies. Applying a room temperature probe had no effect (Figure 3B). Importantly, only a small indentation of the construct was observed in either case (<20 μm). Overall, using a freeze injury to produce a region of cell death within the construct gave consistent results.

**Figure 4:**
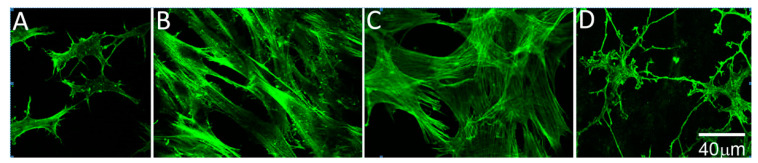
High magnification images of f-actin, 4 days after freeze injury in standard compressed matrices. (**A**) Migrating cells in basal media have a more stellate morphology and rarely exhibit stress fibers; (**B**) Following culture in 10% FBS, migrating cells become fibroblastic, as indicated by the development of a bipolar morphology and stress fiber formation; (**C**) Following culture in TGFβ1, cells developed a broader morphology and prominent intracellular stress fibers were observed; (**D**) Migrating cells in PDGF appeared more elongated with branching, dendritic cell processes.

To assess the effects of growth factors on cell migration through compressed matrices, freeze injuries were produced and the buttons were cultured in serum-free media supplemented with either 10% FBS, TGFβ1, PDGF, or no growth factor (control). Previous studies using other culture models have demonstrated that PDGF stimulates keratocyte spreading and migration, serum induces a fibroblastic phenotype, and TGFβ causes myofibroblast transformation [20,33,37,43,44]. Similar effects were found in our model. Cells in serum-free media had a broad cell body and thin dendritic processes, and did not develop stress fibers (Figure 4A). Following culture in 10% FBS, migrating cells become fibroblastic, as indicated by the development of a bipolar morphology and stress fiber formation (Figure 4B). Treatment with TGFβ_1_ induced more prominent intracellular stress fibers and a broader cell morphology, suggesting transformation to a myofibroblast phenotype (Figure 4C). Treatment with PDGF induced elongation of dendritic processes, without inducing significant stress fiber formation (Figure 4D).

**Figure 5:**
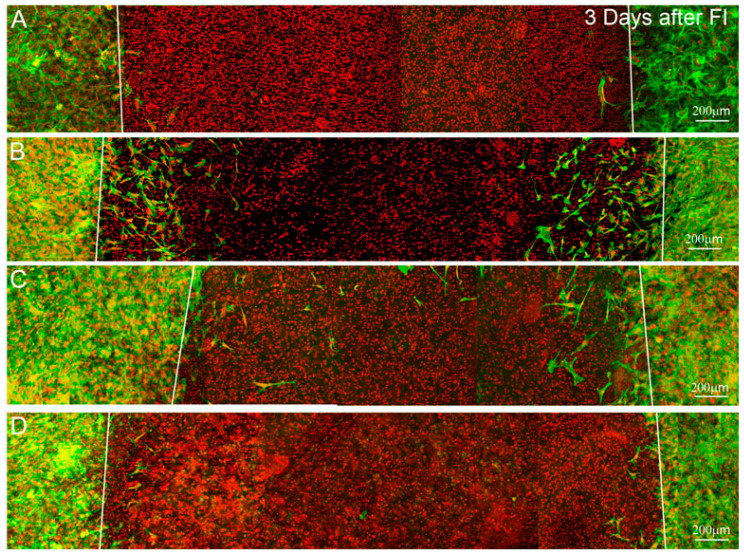
Maximum intensity projection images (~50 microns thick) of f-actin (green) and TOTO-3 (red), 4 days after freeze injury (FI) in standard compressed matrices and culture in (**A**) basal media; (**B**) 10% FBS; (**C**) TGFβ1 or (**D**) PDGF. Migrating cells are observed in the wounded area under 10% FBS and TGFβ1 conditions. However, most of these cells are on or near the surface of the construct. Few migrating cells are observed under serum-free or PDGF culture conditions.

**Figure S1:**
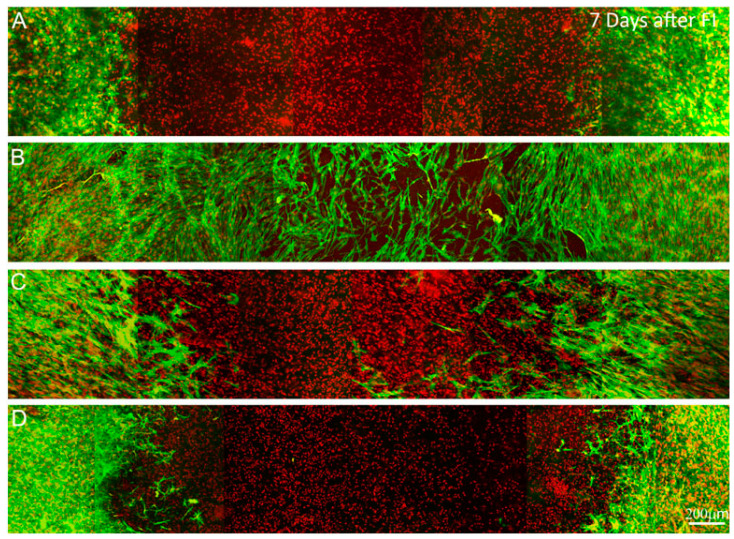
Maximum intensity projection images (~50 microns thick) of f-actin (green) and TOTO-3 (red), 7 days after freeze injury on standard compressed matrices and culture in (**A**) basal media; (**B**) 10% FBS; (**C**) TGFβ1 or (**D**) PDGF. Migrating cells have completely covered the wounded area under 10% FBS conditions. However, most of these cells are on or near the surface of the construct. Few migrating cells are observed under either serum-free or PDGF culture conditions.

We also studied the ability of cells to migrate into the wounded area of the construct. Three days after freeze injury, few migrating cells are observed under serum-free conditions (Figure 5A), or following culture in PDGF (Figure 5D). However, many migrating cells are observed in the wound area following culture in 10% FBS or TGFβ_1_ (Figure 5B,C). Importantly, cells on the top and bottom surfaces of the construct migrated the farthest under all conditions studied, whereas cells inside the construct were unable to migrate effectively through the dense collagen ECM. A similar pattern was observed after 7 days, where cells in serum migrated on top of the matrix to completely cover the wounded area (Supplemental Materials (Figure S1)). Because cells did not migrate effectively inside the dense compressed ECM, this model did not appear to be well suited for studying intrastromal wound healing which is needed to repopulate regions of cell death following PRK or LASIK. However, it should be noted that following PRK surgery, corneal fibroblasts also migrate out of the stroma onto the surface of the photoablated area. During wound healing, increased light scattering is produced both by fibroblasts on the photoablated stromal surface and the activated wound healing keratocytes underlying this area [9,45]. This compressed matrix model may provide an *in vitro* platform for simulating wound healing at the stromal surface.

### 3.2. Sandwiched Compressed Collagen Matrices

To allow more rapid 3-D cell migration, we developed a "sandwich" model in which keratocytes were plated between two acellular collagen matrices prior to compaction. In this model, the keratocytes form a single interconnected layer in between the two compacted collagen layers, similar to keratocyte arrangement in between corneal lamellae. Overall, the keratocytes plated between the collagen layers were evenly distributed and viable. The top and bottom matrices remained firmly attached to one another, even after punching out the buttons with the trephine. Because the buttons were not constrained or under tension, there were some undulations in the matrix. Another limitation was that there were occasional fluid pockets inside the constructs which had to be avoided. 

**Figure 6:**
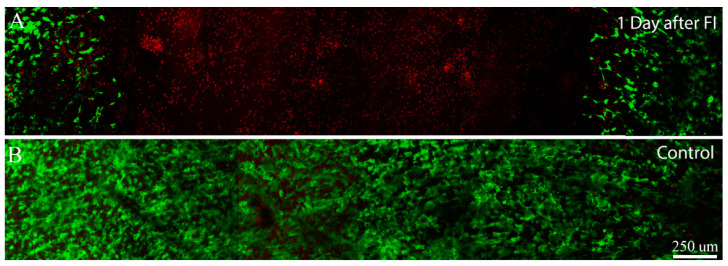
Maximum intensity projection images (~50 microns thick) of Live/Dead staining after 1 day of culture following freeze injury using sandwich construct. Live cells are labeled green and dead cells are labeled red. (**A**) 1 day after freeze injury, induced by pushing on the surface of the matrix using a cold 3 mm diameter probe; (**B**) 1 day control sample, in which a room temperature probe was used.

Following freeze injury, a region of cell death was observed in the center of the constructs, whereas the surrounding cells remained viable (Figure 6A). Applying a room temperature probe had no effect on the keratocytes (Figure 6B). These responses are similar to those observed using standard compressed ECM.

**Figure 7:**
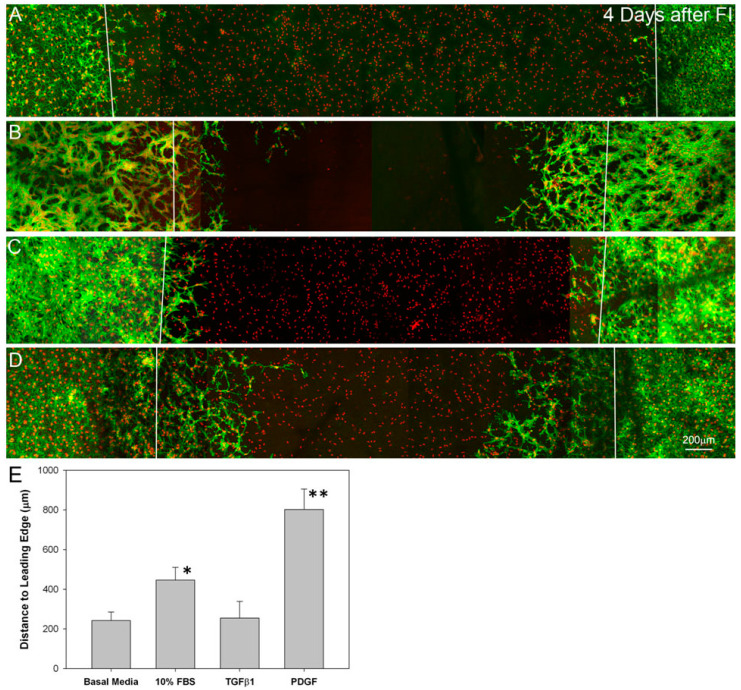
Maximum intensity projection images of f-actin (green) and toto (red), 4 days after freeze injury (FI) on sandwiched matrix constructs, and culture in basal media (**A**); 10% FBS (**B**); TGFβ (**C**) or PDGF BB (**D**); Migrating cells are observed in the wounded area under PDGF (**D**) and 10% FBS (**B**) conditions. Few migrating cells are observed under serum-free (**A**) or TGFβ1 (**C**) culture conditions. E. Quantitative analysis of the distance cells traveled into the injury. Traveling the farthest were cells in PDGF (** P < 0.001 as compared to the other three conditions) and 10% FBS (* P < 0.01 as compared to basal media and TGFβ1).

**Figure 8:**
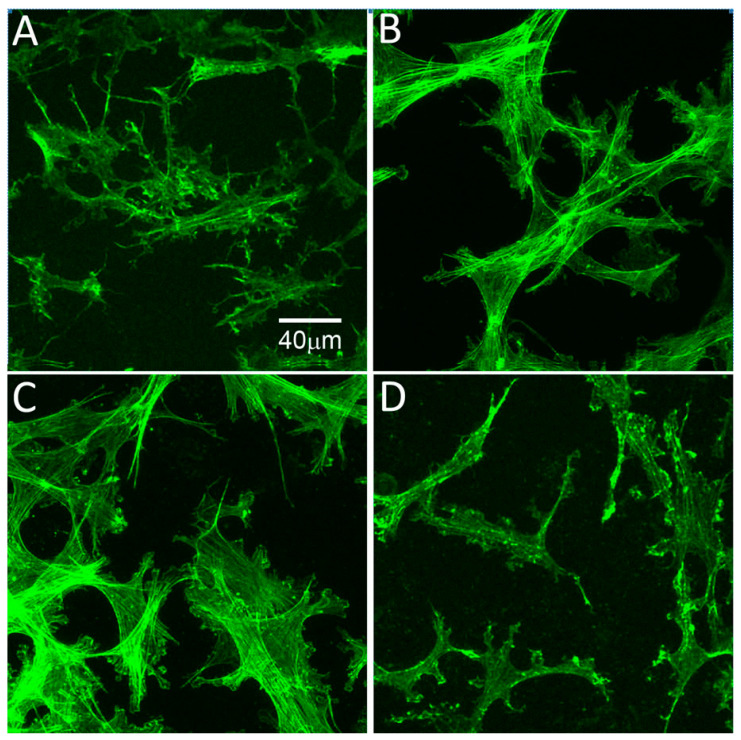
High magnification images of f-actin, 4 days after freeze injury using sandwiched matrix construct. (**A**) Migrating cells in basal media have a more stellate morphology and rarely exhibit stress fibers; (**B**) Following culture in 10% FBS, migrating cells become fibroblastic, as indicated by the development of a bipolar morphology and stress fiber formation; (**C**) Following culture in TGFβ1, cells developed a broader morphology and prominent intracellular stress fibers were observed; (**D**) Migrating cells in PDGF appeared more elongated with branching, dendritic cell processes.

Four days after freeze injury, few migrating cells are observed under serum-free conditions (Figure 7A), or following culture in TGFβ1 (Figure 7C). However, PDGF and 10% FBS showed an increased number of live cells in the "injured" region, suggesting activation and migration of cells from the surrounding area (Figure 7B and D). Orthogonal projections confirmed that cells were migrating in between the collagen layers, and did not enter the matrices (Supplemental Video 1). Migrating cells in serum-free media between compressed matrices had a broad, convoluted cell body and numerous thin dendritic processes, which extended in all directions from the cell; F-actin was generally limited to the cell cortex, and stress fibers were not detected, consistent with that of quiescent corneal keratocytes *in vivo* (Figure 8A). Following culture in 10% FBS, migrating cells developed a bipolar morphology and expressed intracellular stress fibers (Figure 8B). Following culture in TGFβ_1_, cells developed a more spread morphology with prominent stress fibers (Figure 8C). In contrast, treatment with PDGF induced elongation of corneal keratocytes and development of less convoluted cell morphology, with increased ruffling and only occasional stress fibers (Figure 8D). Overall, these growth factor responses are consistent with previous observations within 3D uncompressed collagen matrices, in which serum and TGFβ up-regulate cell contractility and cell induced matrix reorganization, whereas PDGF stimulates cell spreading while maintaining a more quiescent mechanical phenotype [3,37]. In this study, the initial cell seeding density used for the migration studies produced a highly confluent layer. However, similar changes in cell morphology and cytoskeletal organization were observed using a lower plating density in which cells were not interconnected (not shown). To quantify cell migration, constructs cultured for 4 days were fixed and stained and the distance cells migrated into injury was measured. The distance traveled by cells in PDGF (Figure 7E) was larger than the other conditions evaluated (P < 0.001), suggesting that this inter-lamellar migration did not require generation of large cellular forces.

## 4. Conclusions

Overall, these results provide new insights into the interplay between growth factor signaling, cell mechanics and matrix structure. Serum and TGFβ stimulated keratocyte conversion to contractile fibroblast and myofibroblast phenotypes, respectively, in both the standard compressed matrices and the sandwich matrix models. In contrast, PDGF BB induced keratocyte spreading with maintenance of a less contractile, more dendritic phenotype. Following freeze injury in standard compressed matrices, cells did not migrate effectively within the ECM under any condition studied, presumably due to the steric hindrance of the compacted, randomly arranged collagen. However, some cells migrated onto the top of the matrix, consistent with keratocyte healing following PRK. Contractile cells (in serum or TGFβ1) most readily migrated over the matrices to cover the wounded area, whereas non-contractile cells (in basal media or PDGF) did not cover the surface. Our unique “sandwich” model was designed to better mimic the lamellar architecture of the cornea. While significant migration was observed within the construct under all conditions studied, migration was maximal following culture in PDGF, suggesting that inter-lamellar migration can occur without the generation of large cellular forces. From a clinical standpoint, it is preferable to minimize cellular force generation and fibrosis during stromal repopulation following PRK, LASIK or UV cross-linking of the cornea. This model provides an *in vitro* platform for simulating 3-D intra-stromal migration, and for investigating how various wound healing cytokines and their downstream signaling pathways may impact this process. 

## Supplemental Materials

Movie S1 Maximum intensity projection images of f-actin (green) and toto (red), over a range of projection angles on sandwiched matrix constructs. Constructs were cultured for 4 days in PDGF after freeze injury (FI). Image shows cells near the leading edge of the migratory front. MOV-Document (MOV, 374 KB)
