# Correction: Petroll et al. Experimental Models for Investigating Intra-Stromal Migration of Corneal Keratocytes, Fibroblasts and Myofibroblasts. *J*. *Funct. Biomater.* 2012, *3*, 183–198

**DOI:** 10.3390/jfb15070182

**Published:** 2024-07-02

**Authors:** Walter Matthew Petroll, Neema Lakshman, Lisha Ma

**Affiliations:** Department of Ophthalmology, University of Texas Southwestern Medical Center, 5323 Harry Hines Boulevard, Dallas, TX 75390, USA

## Error in Figure

In the original publication [1], there was a mistake in Figure 6 as published. In the montage image of the control (Figure 6B), a figure section was accidentally duplicated. The corrected Figure 6 appears below. New images for both Figure 6A,B were generated from a different experiment to ensure rigor and demonstrate reproducibility. The authors state that the scientific conclusions are unaffected. This correction was approved by the Academic Editor. The original publication has also been updated.

## Figures and Tables

**Figure 6:**
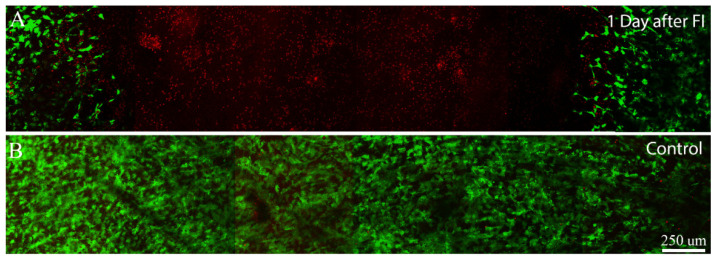
Maximum intensity projection images (~50 microns thick) of Live/Dead staining after 1 day of culture following freeze injury using sandwich construct. Live cells are labeled green and dead cells are labeled red. (**A**) 1 day after freeze injury, induced by pushing on the surface of the matrix using a cold 3 mm diameter probe; (**B**) 1 day control sample, in which a room temperature probe was used.

## References

[1] Petroll W.M., Lakshman N., Ma L. (2012). Experimental Models for Investigating Intra-Stromal Migration of Corneal Keratocytes, Fibroblasts and Myofibroblasts. J. Funct. Biomater..

